# Health Tract, No. 130

**Published:** 1863-04

**Authors:** 


### WOOLEN CLOTHING.

The most healthful clothing for our climate, the year round, is that made of wool. If worn next the skin by all classes, in summer as well as winter, an incalculable amount of coughs, colds, diarrheas, dysenteries and fevers would be prevented, as also many sudden and premature deaths from croup, diphtheria and lung diseases. Winter maladies would be prevented by the ability of a woolen garment to keep the natural heat about the body more perfectly, instead of conveying it away as fast as generated, as linen and flaxen garments do; as also cotton and silk, although these are less cooling than Irish linen, as any one can prove by noticing the different degrees of coldness on the application of a surface of six inches square of flannel, cotton and linen to the skin, the moment the clothing is removed. The reason is, that wool is a bad conductor of heat, and linen is a good conductor.

It is more healthful to wear woolen next the skin in summer, because it absorbs the moisture of perspiration so rapidly, as to keep the skin measurably dry all the time. It is curious to notice that the water is conveyed by a woolen garment from the surface of the body to the outer side of the garment, where the microscope shows it condensed in millions of pearly drops; while it is in the experience of the observant, that if a linen shirt becomes damp by perspiration, it remains cold and clammy for a long time afterwards; and unless removed will certainly cause some bodily ailment.

In the night-sweats of consumption, or of any debilitated condition of the system, a woolen flannel night-dress is immeasurably more comfortable than cotton or linen, because it prevents that sepulchral dampness and chilliness of feeling, which are otherwise inevitable.

The British government make it imperative that every sailor in the navy shall wear woolen flannel shirts in the hottest climates. The shrinkage of woolen garments in washing, whereby they become hard, impervious and board-like, has prevented their more general use; but there are three ways of preventing this, to a greater or less extent; either let about one fourth of the material be made of cotton; have it dyed red or some other color before it is woven; or if it is greatly preferred that it shall be white, exercise proper care in the process of washing. To prevent white woolen stockings from shrinking, have wooden stretchers made of the size and general shape of the foot, and let the stockings remain on them until perfectly dried; or, before rinsing the stocking, double it so as to fold at the heel and lay the foot on the leg, then roll it tight, and ring it crosswise.

In washing all woolen garments, put them in very hot soapsuds-water, so as to be covered; then, when cool enough to allow the hands to be put in, simply press it about with the fingers or hands, and before taking the garment out, make the water for rinsing several degrees hotter than that from which it is to be taken, but instead of wringing the water out, or twisting it about in the water, raise the garment out of the water, up and down a good many times, and then lay it over a line and let it drip dry; this process will, to a considerable extent, prevent fulling or shrinkage, and is worthy of being communicated to every person who expects to be a housekeeper.

